# Investigation of the Quantity of Exhaled Aerosols Released into the Environment during Nebulisation

**DOI:** 10.3390/pharmaceutics11020075

**Published:** 2019-02-12

**Authors:** James A. McGrath, Andrew O’Sullivan, Gavin Bennett, Ciarraí O’Toole, Mary Joyce, Miriam A. Byrne, Ronan MacLoughlin

**Affiliations:** 1 School of Physics & Centre for Climate and Air Pollution Studies, Ryan Institute, National University of Ireland Galway, Galway H91 CF50, Ireland; C.OTOOLE9@nuigalway.ie (C.O.); miriam.byrne@nuigalway.ie (M.A.B.); 2 Aerogen, IDA Business Park, Dangan, Galway H91 HE94, Ireland; andrewosullivanjr@gmail.com (A.O.); GBennett@aerogen.com (G.B.); MJoyce@aerogen.com (M.J.); RMacLoughlin@aerogen.com (R.MACL)

**Keywords:** nebuliser, exhaled aerosol, fugitive emissions, secondary exposure, aerosol, inhalation therapy

## Abstract

Background: Secondary inhalation of medical aerosols is a significant occupational hazard in both clinical and homecare settings. Exposure to fugitive emissions generated during aerosol therapy increases the risk of the unnecessary inhalation of medication, as well as toxic side effects. Methods: This study examines fugitively-emitted aerosol emissions when nebulising albuterol sulphate, as a tracer aerosol, using two commercially available nebulisers in combination with an open or valved facemask or using a mouthpiece with and without a filter on the exhalation port. Each combination was connected to a breathing simulator during simulated adult breathing. The inhaled dose and residual mass were quantified using UV spectrophotometry. Time-varying fugitively-emitted aerosol concentrations and size distributions during nebulisation were recorded using aerodynamic particle sizers at two distances relative to the simulated patient. Different aerosol concentrations and size distributions were observed depending on the interface. Results: Within each nebuliser, the facemask combination had the highest time-averaged fugitively-emitted aerosol concentration, and values up to 0.072 ± 0.001 mg m^−3^ were recorded. The placement of a filter on the exhalation port of the mouthpiece yielded the lowest recorded concentrations. The mass median aerodynamic diameter of the fugitively-emitted aerosol was recorded as 0.890 ± 0.044 µm, lower the initially generated medical aerosol in the range of 2–5 µm. Conclusions: The results highlight the potential secondary inhalation of exhaled aerosols from commercially available nebuliser facemask/mouthpiece combinations. The results will aid in developing approaches to inform policy and best practices for risk mitigation from fugitive emissions.

## 1. Introduction

Aerosol therapy is a mainstay of drug administration in both home and clinical settings for acute and long-term treatments. A variety of aerosol generation technologies deliver drugs to the lungs through patient interfaces across the full range of neonatal to adult patients. The size distributions of generated medical aerosols are typically 1–5 μm [1]. When released indoors, particles can remain airborne for times ranging from minutes to hours [2,3,4].

Exhaled aerosol is aerosol that may have been inhaled but did not deposit on the airway surface, or additionally, aerosol generated by the nebuliser during the inspiratory phase which was released before it could be inhaled. This exhaled fraction is regarded as a fugitive dose and is notable when considering possible unintended bystander inhalation, which includes caregivers or family members. Exposure to fugitive emissions generated during aerosol therapy increases the risk of unnecessary inhalation of medication, as well as toxic side effects [5]. However, the fundamental exhalation mechanism for previously-inhaled aerosol droplets during aerosol therapy has not been well described.

Secondary inhalation of medical aerosols is a risk factor for both caregivers and bystanders and has been highlighted as a significant occupational hazard in both clinical and homecare settings [6,7]. Studies have raised concern surrounding potential secondary exposure of healthcare workers to aerosolised pentamidine, Sustained Release Lipid Inhalation Targeting (SLIT) Cisplatin, adeno-associated serotype 2 vector containing cystic fibrosis transmembrane conductance regulator complementary DNA (tgAAVCF) and ribavirin [7,8,9,10]. Respiratory therapists have an increased risk of developing asthma after entering the profession, which may be partially explained by occupational exposure to a range of aerosolised substances [11,12]. In addition, there is growing global concern about acquired antimicrobial resistance (AMI), with 480,000 people developing multi-drug resistant tuberculosis each year [13]; the use of nebulised antibiotics could potentially contribute to AMI among long-term caregivers who may have inhaled fugitive antibiotic-laden aerosol. 

During the severe acute respiratory syndrome (SARS) outbreak, anecdotal reports highlighted that infected persons receiving aerosol therapies may have facilitated the transmission of SARS under certain circumstances [14]. Clinical guidelines recommended that the generation of aerosols should be avoided [15]; these included the use of non-invasive ventilation, high-flow oxygen (>6 L/min), and nebulisers. 

An in-vitro study examining the mechanical ventilation of a patient with/without expiratory filters reported that the drug deposited at the exhaust port without filtering exceeded the filtering case 160-fold. Additionally, it was concluded that greater than 45% of the nominal dose could become ‘second-hand medical aerosol’ [16]. High-definition video imaging studies used previously-inhaled saline aerosol and smoke particles to estimate the dispersion distance of exhaled air for various systems. Exhaled droplets were observed exiting the side vents of a facemask, and substantial exposure to exhaled air was reported to occur within 0.8 m [17]. Nebuliser airflow, lung function and interface type were all reported to impact upon dispersion distance [18,19,20,21]. A limitation of the visualisation approach is that the visible boundaries of exhaled flows could only be used as a guide to the real behaviour of droplets in exhaled air [22].

In the respiratory therapy context, the secondary exposure pathway is not widely understood. Limited evidence exists to explain fugitive emissions, and to date, research has primarily focused on infection control models. The current study aimed to enhance the understanding of secondary exposure to aerosol emitted during respiratory therapy with commonly-used equipment. This study examined the potential for secondary (caregiver/bystander) exposure in domestic and clinical caregiving settings, by examining some key factors that influence secondary exposure: caregiver position and device type. 

## 2. Materials and Methods 

### 2.1. Nebulisers (VMN & JN) & Non-Invasive Ventilation Interfaces

The design and operation features of two prevalent nebuliser technologies were assessed regarding their emission of exhaled aerosol. These were a vibrating mesh (VMN) used in combination with an aerosol chamber (Aerogen Solo/Ultra, Aerogen, Galway, Ireland) and a jet (JN) (Cirrus 2, Intersurgical, Wokingham, United Kingdom), both in clinical use today. This study incorporated two non-invasive ventilation interfaces (facemask and mouthpiece) in combination with the commercially available aerosol generators. The VMN used a valved facemask, while the JN used an open facemask. For the mouthpiece scenarios, both an unfiltered mouthpiece and filtered mouthpiece were used with each setup. A filtered mouthpiece refers to a filter placed on the exhalation port, while an unfiltered mouthpiece refers to the case where no filter is placed on the exhalation port. To replicate the ideal conditions achieved when a patient uses an interface correctly, each mask was glued to a plastic sheet to avoid a leak, while the mouthpieces were attached using a custom fixture. A supplemental gas flow rate of 6 L/min was used with the VMN and the manufacturer-prescribed 8 L/min was used for the JN. 

Each nebuliser was individually assessed under simulated real use conditions. The laboratory room had a volume of 55 m^3^, three internal doors, but no external doors or windows; the room was mechanically ventilated with an air exchange rate of 2.70 h^−1^. The ambient temperature and relative humidity in the test room were typically 22 °C and 35%, respectively, during the test period.

### 2.2. Simulated Patient Breathing

Each aerosol generator/interface combination was connected to a breathing simulator (ASL 5000, Ingmar Medical, Pittsburgh, PA, USA) via an absolute filter (RespirGard II 303, Baxter, Dublin, Ireland). A simulated adult breath was used (breath rate 15 BPM, tidal volume 500 mL and inspiratory: expiratory (I:E ratio) 1:1). As a result, no Institutional Review Board was required for this study. 

### 2.3. Characterisation of Aerosol Dose Distribution

**Figure 1:**
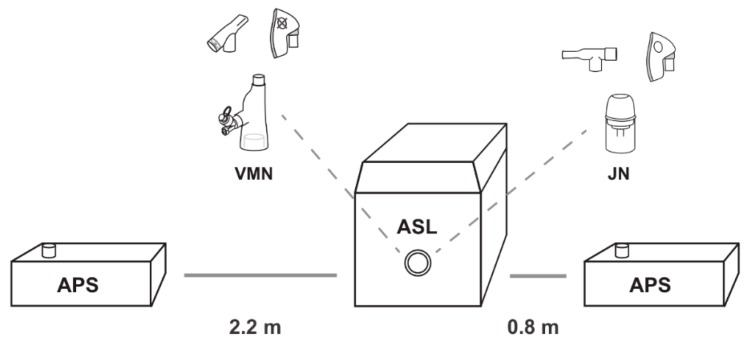
Aerodynamic particle sizer (APS) at varying distances relative to the simulated patient (ASL 5000) with VMN and JN facemask/mouthpiece iterations. VMN, vibrating mesh nebuliser; JN, jet nebuliser; ASL, breathing simulator.

A nominal dose of 2.5 mL (2.5 mg) albuterol sulphate (Ventolin, 1 mg/mL, GSK, Cork, Ireland) was nebulised in each test run. Aerosol delivery performance was evaluated by characterising the inhaled dose, that is to say, drug delivered to the patient. To determine the inhaled dose, each aerosol generator/interface combination was attached to an absolute collection filter (Figure 1). Albuterol is commonly nebulised as a medication, and also as a formulation used in the characterization of aerosol drug delivery systems, and is specified for use as a tracer aerosol [23]. In this study, albuterol was eluted from filter or nebuliser components using a wash buffer. Albuterol mass, expressed as a fraction of the nominal dose, at the end of each run was extracted and quantified using UV spectrophotometry (Biochrom UV Vis, Cambridge, UK) at 276 nm and interpolation on a standard curve of albuterol sulphate. The exhaled dose, the drug that was exhaled by the spontaneously breathing patient, was also characterised (mouthpiece testing only) by placing a collecting filter at the exhalation ports of each nebuliser/mouthpiece combination (Figure 1). Additionally, the residual mass, the drug that was available for nebulisation but remained in the device, was reported for both VMN and JN devices. All testing was carried out in triplicate.

### 2.4. Characterisation of Fugitive Emissions

The primary aerosol monitoring instrument used in this study was an aerodynamic particle sizer (APS) (APS, model 3321 TSI Inc., St. Paul, MN, USA) measuring aerosol size distributions from 0.5 to 20 μm. Throughout the experiments, the aerosol concentrations and size distributions of the aerosol were continuously measured in real time using two APSs, which were located at respective distances of 0.8 m and 2.2 m from the simulated patient. These distances were chosen on the basis that 0.8 m is approximately one arm’s length away, simulating, for example, a caregiver holding a nebuliser/facemask to a patient’s face. A distance of 2.2 m was chosen on the basis that it is approximately the distance between beds in a primary care centre, simulating a patient situated in a bed next to the patient receiving the aerosol therapy. A 5-min baseline level of aerosol was established in the room pre-nebulisation. Nebulisation was then initiated with the APSs recording data for 25 min, and at 20-s intervals. A duration of 25 min was sufficient for nebulising the entire dose with a period for aerosol decay post-dose. This testing was carried out concurrently with the aerosol dose distribution testing described above.

The statistical package IIBM SPSS Statistics 24 (IBM Corp., Armonk, NY, USA, 2013) was used to analyse the data. Summary and descriptive statistics were performed on aerosol concentrations. All data are expressed as mean, minimum and maximum.

## 3. Results

### 3.1. Aerosol Distribution 

The collection filters were analysed in terms of the inhaled dose, exhaled dose and residual mass, and the results are summarised in Table 1. While the residual mass and inhaled dose vary depending on the nebuliser type, results within each nebuliser group are substantially equivalent.

### 3.2. Time-Averaged Aerosol Concentrations 

Prior to nebulisation, the mean and standard deviation of the ambient concentrations were 0.006 ± 0.002 (0.002 to 0.014) mg m^−3^. Table 2 confirms that fugitive emissions were recorded during the nebulisation events for the facemask and unfiltered mouthpiece combinations. There was no recorded increase in aerosol concentration when using the VMN/filtered mouthpiece, while increased aerosol concentrations were recorded for the JN/filtered mouthpiece.

**Figure 2:**
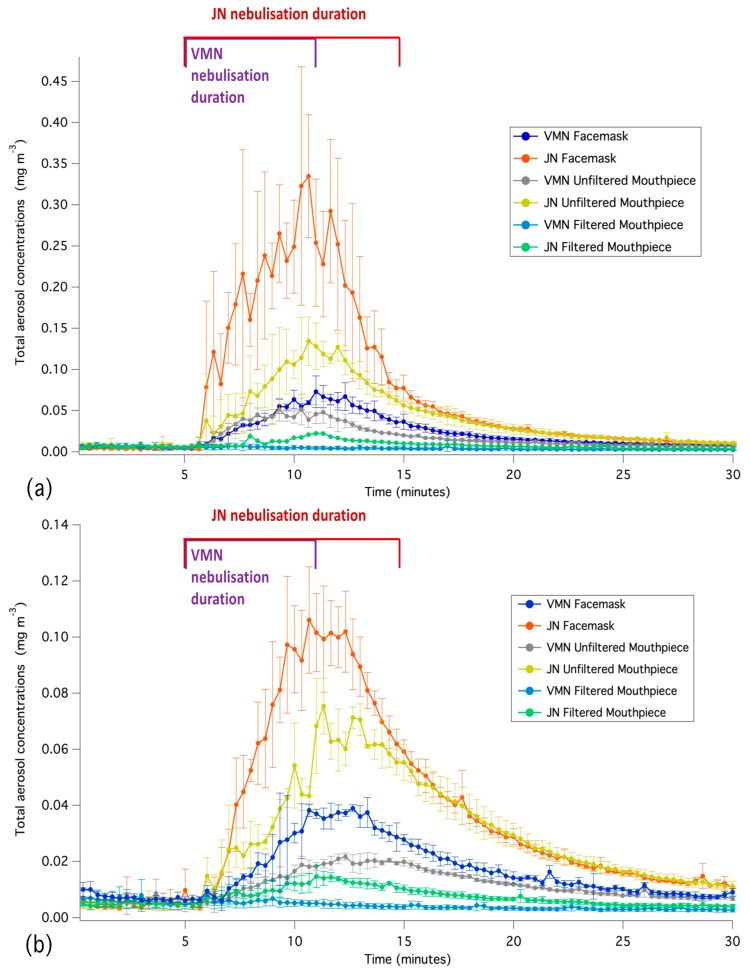
Time-series averaged aerosol concentrations for the three runs recorded at a distance of (**a**) 0.8 m and (**b**) 2.2 m from the different patient interface combinations. The y bars represent one standard deviation at each time step.

Figure 2a compares the aerosol concentrations at the distance of 0.8 m away from the patient interface combinations. The highest concentration is observed at 0.8 m from the facemask/mouthpiece combination, corresponding to the end of nebulisation and due to proximity to the facemask. Once the nebulisation has ceased, uniform concentrations, as measured by the two APSs, are gradually established in the room. 

It can be seen from Table 2 that the use of VMN/unfiltered mouthpiece and JN/unfiltered mouthpiece combinations resulted in reduced exhaled aerosol concentrations compared with the corresponding facemask. 

The time-averaged difference between the exhaled aerosol recorded 2.2 m away from the facemask/mouthpiece combinations compared with the 0.8 m were approximately 49% for the JN/open facemask, 78% for the VMN/valved facemask, 67% for the VMN/unfiltered mouthpiece, and 71% for the JN/unfiltered mouthpiece. Figure 2b compares the aerosol concentrations at the distance of 2.2 m away from the patient interface combinations.

The temporal variations in the aerosol concentrations when using the jet nebuliser resulted in a larger difference between the two APS readings for the open facemask combination compared with the other configurations. The largest variations in aerosol concentrations between the two APSs occurred during the nebulisation period; in all cases, there was less than a 10% averaged difference between the two sets of measurements in the final 15 min after nebulisation, which indicates the diffusion of fugitive emissions throughout the room.

### 3.3. Aerosol Droplet Sizing

Table 3 summarises the averaged MMAD over the 30-min period recorded by the APS 0.8 metres from the facemask/mouthpiece. There is a noticeable shift in the MMAD during the nebulisation event when using the facemask/mouthpiece compared with the initially-generated aerosol. 

The reduced size and restricted opening area of the valved facemask are assumed to result in an exhaled aerosol with a lower MMAD than that exhaled in the open facemask configuration. 

### 3.4. Inhalation Exposure 

The percentage of the original drug that could potentially be inhaled by a caregiver and a bystander was estimated. The averaged JN/open facemask time-series data were selected as these exhibited the highest level of fugitive emission. The five-minute baseline average ambient aerosol concentration was subtracted from the time-series data to focus solely on fugitive emissions. Short-term exposure values for inhalation to represent light activity for the caregiver (1.3 × 10^−2^ m^3^/min) and sedentary activity for the bystander (4.8 × 10^−3^ m^3^/min), both in the age range of 41 to <51 years old, were calculated [24]. The caregiver and bystander were assumed to be at respective distances of 0.8 m and 2.2 m from the patient. In this scenario, the caregiver and bystander were estimated to be exposed to 1.05% and 0.20% of the original nominal drug dose placed in the nebuliser, respectively.

## 4. Discussion

This study highlights the potential secondary inhalation exposure to fugitive emissions for caregivers and other bystanders during a standard nebuliser treatment. This study provides real-time monitoring data of size-resolved indoor aerosol concentrations when an aerosol generator/non-invasive ventilation interface connected to a breathing simulator is used to replicate an adult breathing pattern. While the current approach did not explicitly remove the ambient aerosol contribution from the data, it is evident that the observed changes in aerosol concentrations were due to aerosol from the nebulisation events. 

Several factors influence inhalation dose [5,25,26,27], and all of these are likely to affect the quantity of released aerosol. Environmental factors (including room dimensions and layout, air turbulence, ventilation, and temperature) also affect the released aerosol distribution [28,29,30]. The current observations should therefore be extrapolated with caution, but nevertheless, it can be noted that this study provides compelling evidence that a caregiver or bystander receives an inadvertent drug dose which is intended for the patient.

It can be noted that the respective inhalation exposures for a caregiver (1.05%) and bystander (0.20%) were based on fugitive emissions from a standard treatment using a standard compressor-driven jet nebuliser. However, it is common that higher nominal doses are administered (5–15 mg/h) for periods ranging from 4–8 h [31,32,33]. Aerosolised ribavirin has also been reported to be nebulised at a rate of 6 g over 12–18 h per day [34]. A caregiver/medical practitioner may be working in a hospital where several patients may receive aerosol therapy throughout the day, over many days/weeks or for several years. Therefore, the potential for chronic low dose occupational exposure is of concern [35].

In this study, medical nebulisers generated an aerosol with a size distribution within the respirable range of 1–5 µm. However, the MMAD of the fugitive emissions ranged between 0.860–1.437 µm across all nebuliser combinations, indicating that a considerably smaller aerosol size fraction is released than originally generated by the nebuliser. This is a significant finding, as the lower size fractions remain airborne for extended periods of time [29].

The VMN combinations had reduced fugitive emissions compared with the JN, likely due to the combination of lower airflow and the valve system on the VMN. In both cases, the unfiltered mouthpiece had reduced fugitive emissions compared with the facemask. Since there was no appreciable change in the residual drug analysis for the different facemask/mouthpiece combinations, it can be concluded that the mouthpieces’ shape and size do not influence inertial impaction and deposition [36]. For the filtered mouthpiece combination, while there were some observed fugitive emissions from JN/filtered mouthpiece combination compared with the VMN, it is possible that the higher driving force required for the normal operation of the JN/mouthpiece caused additional system pressure, allowing aerosol to escape through the custom fixture; however, it is unclear if this would be replicated with a human patient. An earlier imaging study observed no emitted droplets when a respiratory filter was placed on the exhalation port [17]. Other research shows that attaching an exhalation filter to the nebuliser was 93% effective in capturing exhaled aerosol droplets [37]. Facemask design may also contribute to this. By necessity, compressor-driven jet nebulisers must be operated with an open facemask; i.e., one without valves. This, in combination with the continual positive pressure air flow, acts to force aerosol out of the device and into the environment throughout both the inhalation and exhalation cycles of the breath. This is not the case with the vibrating mesh nebulisers, where a driving air flow is not required for aerosol generation, and the valve system on the device described aids in building a reservoir of aerosol within the patient interface which is available for the next breath.

In a previous study, the drug distribution between inhalation filters, exhalation filters, ventilator tubing and medication reservoirs was quantified for a VMN and JN [5], and some comparisons are possible with this study. The residual drug in the VMN was 5.2% compared with 5.2–6.8% (mean value for each interface) in the current study, while the residual for the JN was 38.0% compared with 38.4–40.1% in the present study, albeit with a different JN. Inhalation filters recorded 39% and 22% [5], compared with the current study of 46.7–50.3% and 22.0–35.9% for the VMN and JN, respectively.

## 5. Conclusions

In the respiratory therapy context, the secondary exposure pathway is not widely understood. An adult breathing pattern in combination with a facemask/mouthpiece was used to replicate a standard nebuliser treatment and monitor the consequent release of fugitive emissions. To the best of the authors’ knowledge, this is the first study that quantifies time-series aerosol concentrations and the size distribution of fugitive emissions that result from standard nebuliser treatment. The findings confirm a potential exposure risk to caregivers and other bystanders to medical aerosols and highlight that variations in patient interfaces (facemask and mouthpiece) and aerosol generators (VMN and JN) influence fugitively-emitted aerosol concentrations. The purpose of this study is to aid in developing approaches for healthcare organisations to inform policy and best practices for risk mitigation from fugitive emissions. Future studies should focus on investigating these factors in a real-life clinical scenario.

## Figures and Tables

**Table 1:** Inhaled dose, exhaled dose and residual mass recorded during each nebulisation event (using facemask and mouthpiece); mean and standard deviations over the three events (results expressed as a percentage of the nominal dose placed in the nebuliser).

Nebuliser/Interface	Residual Mass	Inhaled Dose	Exhaled Dose
VMN/valved facemask	6.87 ± 1.43%,	46.69 ± 3.06%	N/A
JN/open facemask	39.03 ± 2.85%	21.95 ± 0.66%	N/A
VMN/unfiltered mouthpiece	6.49 ± 1.11%	49.46 ± 3.17%	N/A
JN/unfiltered mouthpiece	40.10 ± 5.49%	29.22 ± 1.71%	N/A
VMN/filtered mouthpiece	5.26 ± 2.04%	50.32 ± 1.90%	14.53 ± 7.85%
JN/filtered mouthpiece	38.43 ± 1.57%	35.94 ± 1.81%	29.45 ± 1.41%

**Table 2:** Thirty-minute averaged aerosol concentration during each nebulisation event (using different facemasks and mouthpieces); mean and standard deviations over the three events.

Nebuliser Type	Facemask (mg m^−3^)	Unfiltered Mouthpiece (mg m^−3^)	Filtered Mouthpiece (mg m^−3^)
Jet Nebuliser	0.072 ± 0.001	0.039 ± 0.004	0.009 ± 0.001
Vibrating Mesh Nebuliser	0.022 ± 0.001	0.017 ± 0.002	0.004 ± 0.001

**Table 3:** Thirty-minute averaged aerosol concentration during each nebulisation event (using different facemasks and mouthpieces); mean and standard deviations over the three events.

Nebuliser Type	Facemask (µm)	Unfiltered Mouthpiece (µm)	Filtered Mouthpiece (µm)
Jet Nebuliser	1.338 ± 0.098	1.267 ± 0.07	1.155 ± 0.081
Vibrating Mesh Nebuliser	1.160 ± 0.205	0.890 ± 0.044	1.432 ± 0.241
